# Mystifying Fibroepithelial Stromal Polyps of the Breast Nipple: A Report of Three Rare Cases and a Literature Review

**DOI:** 10.7759/cureus.88567

**Published:** 2025-07-23

**Authors:** Permeet K Bagga, Loveleen Kaur, Gulwinder Kaur, Poonam Kattru

**Affiliations:** 1 Department of Pathology, Government Medical College, Amritsar, Amritsar, IND

**Keywords:** breast nipple growth, histopathology, nipple acrochordon, nipple fibroepithelial stromal polyp, pedunculated nipple polyp

## Abstract

Fibroepithelial stromal polyps (FEPs) or acrochordons of the breast nipple are seldom encountered benign tumors with mesoderm origin. These FEPs are frequently reported along the skin folds in the form of either skin-colored, tan, or hyperpigmented pedunculated or sessile papule-like or polypoidal growths. Three adult female patients presenting to a tertiary care hospital underwent surgical excision of nipple growth. All three cases had local presentation ranging from asymptomatic growth to one with tenderness alone and another with tenderness, pruritis, and scant nipple discharge. Although all three had varied provisional clinical diagnoses, after histopathological examination, the final diagnosis of FEP of the breast nipple was confirmed in all three cases. FEPs of nipple share histomorphological features with FEPs occurring at other common sites, with variably cellular, abundant fibrovascular and collagenous stroma covered by epithelium lining. The presence of nuclear pleomorphism and increased mitosis can lead to significant confusion with benign and malignant primary breast pathologies. On six months post-excision follow-up, none of the three patients reported any fresh complaints. The findings of this short case series highlight the need for awareness, the importance of early diagnosis, and potential clinical mimics in these cases.

## Introduction

Fibroepithelial stromal polyp (FEP) or the acrochordon is quite a well-known entity of mesodermal origin and overall benign course [1]. FEP can be solitary or multiple, routinely with maximum dimension reaching up to a few millimetres (mm), and are encountered at anatomical locations including skin folds of the neck, axilla, perineum, and eyelids [2]. As opposed to these common sites, lesser-known sites include the male penis, urethra, and female labia [3,4]. Breast nipple FEPs are almost never heard of in day-to-day practice, with only about 10 case reports in the past [5]. These are reported mostly among females of reproductive age groups. The nipple FEP is identified as a variable-sized, skin-coloured or hyperpigmented, exophytic smooth or cauliflower-like lesion arising from the nipple, with or without an evident stalk [6,7].

We present the largest single-centre encounter of rare nipple FEPs diagnosed in three adult females with varying clinical presentations.

## Case presentation

Clinical presentation and examination

Case 1

**Figure 1:**
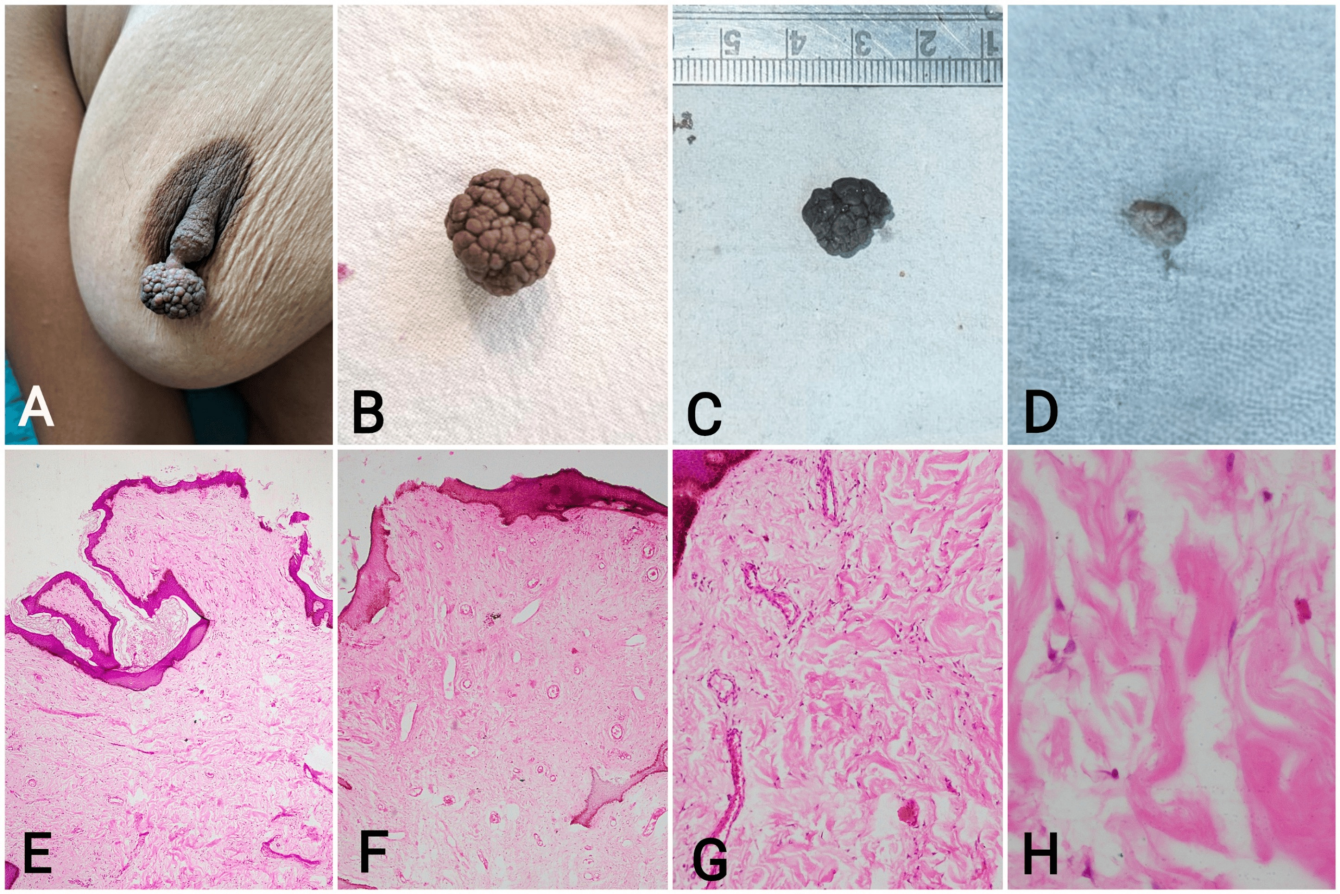
Photographs showing a) the clinically evident polypoid growth of the breast nipple in a 30-year-old female; b-d) excised gross nipple exophytic growth specimens from the three cases. Microphotographs showing e-f) polypoid tissue lined by focally hyperplastic stratified squamous epithelium with underlying subepithelium showing fibrovascular core and abundant areas of collagenization (H&E, x40); g) minimal perivascular chronic lymphocytic inflammation within the subepithelium (H&E, x100); h) high-power showing thick collagen bundles and interspersed stromal stellate cells (H&E, x400).

A 30-year-old, P2L2 female hailing from a rural area presented with a complaint of a single, smooth, hyperpigmented, hanging lesion over the nipple of the right mammary region for the past 18 years (Table 1, Figure 1A). Her last childbirth was five years ago, and three years ago, she ceased breastfeeding. There were no associated local or systemic significant complaints reported by the patient. The lesion was surgically excised with a provisional clinical diagnosis of breast dermatofibroma. Macroscopic examination of the specimen received for histological examination showed a hyperpigmented, globular, soft tissue piece with a convoluted external surface (Figure 1B) and smooth, homogenous grey-yellow cut surface.

**Table 1:** Comparison of the clinical presentations and gross findings of the three fibroepithelial stromal polyp (FEP) cases.

Case	Age (years)	Parity	Laterality of affected breast nipple	Duration (years)	Provisional clinical diagnosis	Number of nipple growth (s)	Size of nipple growth	Pigmentation status of nipple growth	Other local complaint(s)
1	30	Multi-parous	Right	18	Dermatofibroma	Single	2x1.5x1.5cm	Increased	None
2	18	Nulliparous	Left	10	Papilloma	Single	1.4x1x0.5cm	Increased	Pruritis, Tenderness, Nipple discharge.
3	31	Multi-parous	Left	0.5	Nipple adenoma	Single	0.5x0.3x0.3cm	No increase	Tenderness

Case 2

We describe the case of an 18-year-old, nulliparous female presenting with a decade-long history of left nipple growth. This growth was insidious in onset and very slowly progressing in size. Over the past years, no local complaints were experienced by the patient; however, over the past two months, she started experiencing on-and-off itching and nipple tenderness. Occasional small volume colourless, thin, blood-tinged serous nipple discharge was also documented. On local examination, a single greasy left nipple growth was observed (Table 1, Figure 1C). There was no increase in pigmentation or skin ulceration. Clinically, no palpable regional lymph nodes were detected. She underwent surgical excision of the growth with clinical suspicion of papilloma.

Case 3

A 31-year-old, P3L3 female presented with the complaint of a smooth, brown-coloured, pedunculated growth at the right nipple region for the past six months (Table 1). Local examination confirmed a small skin-coloured growth over the right nipple, which was tender on touch (Table 1, Figure 1C-1D). No discharge or local rise of temperature was noted on palpation. On palpation, no breast lump, nodule, or clinically evident lesion was noted in bilateral breasts. There was no palpable axillary or cervical lymphadenopathy. The provisional clinical diagnosis in the case was nipple adenoma.

Personal and family histories

On general physical examination, all three cases were moderately nourished and built with regard to their age and gender. They all had good nutritional status and were immunocompetent. None of the cases had any concurrent systemic lesion or complaint. No significant finding was revealed on the survey of their past and personal history. None of them reported the presence of diabetes, hypertension, dyslipidemia, or the familial history of any event of breast, colorectal, or genitourinary cancer.

Laboratory investigations and histopathological examination

The haematological, coagulation, and serum investigations were within normal range for all three cases. The viral markers status for hepatitis C virus, human immunodeficiency virus, and hepatitis B antigen were non-reactive in all. Radiological investigation - sonomammography - was not performed in any of the cases.

The excised nipple growth specimens from all three cases were sent for histopathological examination by immersing in an adequate volume of 10% neutral buffered formalin. The external surface appeared hyperpigmented in two cases and normopigmented in the third. The outer surface of all the specimens was rubbery to touch and firm in consistency (Figure 1). On sectioning, the inner surface in all three cases was solid and homogenously grey-white.

Hematoxylin-and-eosin-stained microscopic slides examination of all three cases revealed polypoid tissue lined by focally hyperplastic stratified squamous epithelium with areas of loss of rete ridges. Underneath the lining epithelium, abundant fibrocollagenous and fibrovascular subepithelium showed the presence of occasional spindle-shaped cells, dense collagen, and scant to mild perivascular lymphocytic inflammation (Figure 1E-1H). No fascicular or storiform arrangement of smooth muscle bundles was identified microscopically. No obvious lactiferous ducts or sweat glands were noted. In addition, there was no evidence suggestive of malignancy or atypical mitotic activity. With this histomorphological picture, a final diagnosis of FEP of the nipple was given in all three cases.

The post-excision recovery was uneventful, and at six months follow-up, none of the patients reported any growth recurrence or fresh complaints.

## Discussion

FEPs or acrochordons are benign tumours of mesenchymal and ectodermal origins known to develop as small growths along sites of skin folds such as neck, oral cavity, eyelids, inframammary, and a few at genitourinary area [8]. Breast nipple FEPs have an extremely rare incidence with only about 10 isolated case reports to date [7]. Although the exact underlying etiopathogenesis is still obscure to date, FEPs are proposed to arise either secondary to focal elastic tissue loss or as a slow-growing hamartoma or fibroma that encompasses multifarious tissues [9, 10].

The literature suggests a likely association between FEP development and systemic conditions like obesity, endocrine pathologies including diabetes mellitus, and insulin resistance, and some evidence of carbohydrate and lipid metabolism disorders has been implicated to play a role in FEP occurrence. Female gender, genetic predisposition, pregnancy status, hormonal changes, and human papilloma virus serotypes 6 and 11 (HPV-6, 11) may also likely play a role in the development and progression of the FEPs [11-13]. The three cases studied revealed no personal history of any systemic disorder or long-standing medical or surgical condition. The family histories were also negative for dyslipidemia and diabetes.

An interesting observation made during the study was the long duration between the first observation of the nipple lesion by the patient and its final surgical excision. This could be due to interplay by a myriad of reasons, including but not limited to a gradual slow progression of the lesion, absence of any associated loco-regional symptoms, and possible financial and cultural barriers.

As reported previously in the literature, the occurrence of FEPs is usually beyond the fourth decade of life [14]; however, in our short case series, all the cases belonged to much younger ages of 18, 30, and 31 years. Two of the three cases were multiparous, while only one was nulliparous. These may suggest the possible impact of hormone changes and gestation in FEP pathogenesis. The development of associated local complications observed in two of three cases in this study, namely, tenderness, pruritis, and discharge, can be due to due to microtrauma and possible intermittent torsion of the stalk, as has been suggested in the previously reported studies [7,12,15,16].

The majority of FEPs are encountered clinically in the form of small smooth pedunculated or sessile growths, measuring merely a few millimetres in size, with occasional larger FEPs. The largest reported case of FEP to date is of a 42-cm-long groin FEP [17]. Nipple FEPs are frequently discovered early with small lesion size, probably due to easy detection and/or sudden development of local effects if the stalk undergoes torsion or infarction [12].

Representative lesion biopsy followed by generous, unbiased histopathological examination is mandatory for arriving at the definitive diagnosis of these rare entities. Examination of nipple FEP reveals its shared histomorphological characteristics as FEPs arising at other commoner locations. The lesions are polypoid and lined by attenuated or unremarkable stratified squamous epithelium with abundant underlying stroma with fibrovascular core. The stroma can display variable cellularity ranging from hypocellular with abundant collagenous matrix and only a few bland spindle-shaped cells with indistinct cytoplasm, or hypercellular with the presence of numerous stellate and multinucleated cells, along with cells showing nuclear pleomorphism, atypia and increased mitosis [5,12].

The importance of histopathological examination of these rare nipple FEPs cannot be undermined as they clinically mimic breast pathologies including pseudoangiomatous stromal hyperplasia, mammary fibromatosis, and malignant metaplastic breast carcinoma [18]. Moreover, previous literature reports the unfortunate development of malignancy within FEPs, including basal cell carcinoma and squamous cell carcinoma [19, 20].

Treatment modalities for smaller FEPs using either cryotherapy or cauterisation are the norm, while surgical excision is the only definite treatment option for larger lesions to date. Although benign, FEPs display the property of local recurrence if not completely excised. This underscores the need for ensuring a surgical safety margin of at least a few millimetres [2].

## Conclusions

FEPs are benign mesodermally derived pathologies that can extremely rarely be encountered at the breast nipple. With only a handful of cases in the literature, FEPs should be considered as a clinical differential in all reproductive-age-group individuals presenting with slow-growing nipple growth. It can go undisclosed for prolonged periods due to its asymptomatic behaviour and be only brought to clinicians’ attention when the patient develops complications. As such, the patient can go extended duration of time before it is surgically excised.

The macroscopic and microscopic appearance of nipple FEPs can be troublesome in cases where gross secondary changes, microscopic cellular atypia, and hypercellularity are encountered. However, its benign and malignant mimics as well as the rare development of carcinoma within FEP should always be cautiously ruled out with a microscopically clear surgical resection margin status. For cases with ambiguous histopathological features, the role of immunohistochemistry markers should not be forgotten.
